# Remodeling of the Inner Hair Cell Microtubule Meshwork in a Mouse Model of Auditory Neuropathy AUNA1

**DOI:** 10.1523/ENEURO.0295-16.2016

**Published:** 2016-12-29

**Authors:** Clément Surel, Marie Guillet, Marc Lenoir, Jérôme Bourien, Gaston Sendin, Willy Joly, Benjamin Delprat, Marci M. Lesperance, Jean-Luc Puel, Régis Nouvian

**Affiliations:** 1 Inserm U1051, Institute for Neurosciences of Montpellier, University of Montpellier, 34295 Montpellier, France; 2 Division of Pediatric Otolaryngology, Department of Otolaryngology-Head and Neck Surgery, University of Michigan Health System, Ann Arbor, MI 48109

**Keywords:** cochlea, deafness, diaphanous, diap3, cuticular plate

## Abstract

Auditory neuropathy 1 (AUNA1) is a form of human deafness resulting from a point mutation in the 5′ untranslated region of the *Diaphanous homolog 3* (*DIAPH3*) gene. Notably, the *DIAPH3* mutation leads to the overexpression of the DIAPH3 protein, a formin family member involved in cytoskeleton dynamics. Through study of diap3-overexpressing transgenic (Tg) mice, we examine in further detail the anatomical, functional, and molecular mechanisms underlying AUNA1. We identify diap3 as a component of the hair cells apical pole in wild-type mice. In the diap3-overexpressing Tg mice, which show a progressive threshold shift associated with a defect in inner hair cells (IHCs), the neurotransmitter release and potassium conductances are not affected. Strikingly, the overexpression of diap3 results in a selective and early-onset alteration of the IHC cuticular plate. Molecular dissection of the apical components revealed that the microtubule meshwork first undergoes aberrant targeting into the cuticular plate of Tg IHCs, followed by collapse of the stereociliary bundle, with eventual loss of the IHC capacity to transmit incoming auditory stimuli.

## Significance Statement

The mutation in the *Diaphanous homolog 3* gene, which leads to overexpression of diap3 protein, underlies the human deafness called auditory neuropathy 1 (AUNA1). Although diap3 is known to regulate the cytoskeleton, the signaling cascade operating in AUNA1 is still unclear. Using a transgenic mouse model of AUNA1, which overexpresses diap3, we show that microtubules accumulate at the apical pole of the auditory sensory cells, the inner hair cells. The microtubule network remodeling is followed by the anatomical alteration of the mechanotransduction apparatus, which could explain the failure to transduce acoustic stimuli into neural message. Altogether, this study suggests that a massive microtubule remodeling occurs in the mouse model of AUNA1.

## Introduction

Auditory neuropathy is a form of human deafness in which the auditory brainstem response (ABR) is absent or altered, while outer hair cells (OHCs), which amplify the sound stimulation in the cochlea, are still preserved (Starr et al., 1996; Rance and Starr, 2015). A variety of etiologies may result in this disorder, including defects in cochlear inner hair cells (IHCs) that transduce sound stimulation into neurotransmitter release, and defects or absence of the auditory afferent fibers that convey the neural message to the cochlear nuclei. Auditory neuropathy may be a systemic condition involving neuropathies of multiple cranial and peripheral nerves, whereas nonsyndromic auditory neuropathy is limited to the auditory nerve. At this time, most cases of nonsyndromic auditory neuropathy arise from synaptic transfer failure (Moser and Starr, 2016).

A mutation in the *Diaphanous homolog 3* (*DIAPH3*) gene is responsible for autosomal dominant nonsyndromic auditory neuropathy 1 (AUNA1; (Greene et al., 2001; Kim et al., 2004; Starr et al., 2004). DIAPH3 belongs to the formin-related family, known to promote the nucleation and elongation of actin filaments and to stabilize microtubules (Wallar and Alberts, 2003; Higgs, 2005; Kovar, 2006). Strikingly, the point mutation in the 5′ untranslated region of the human *DIAPH3* leads to overexpression of the DIAPH3 protein (Schoen et al., 2010). Accordingly, a *Drosophila* model that expresses a constitutively active *diaphanous* protein in the auditory organ exhibits an impaired response to sound (Schoen et al., 2010). Transgenic (Tg) mice overexpressing *diap3* (the murine ortholog of *DIAPH3*) have been an useful tool to dissect the AUNA1 mechanism (Schoen et al., 2013), demonstrating that overexpression of diap3 in Tg mice recapitulates the human AUNA1 phenotype, i.e., a delayed-onset and progressive hearing loss leaving OHCs unaffected (Schoen et al., 2013). In addition, IHCs of Tg mice show fusion of the stereociliar bundle, implicated as the primary cause of the deafness (Schoen et al., 2013). However, the molecular mechanisms responsible for these morphological changes are still unknown.

Here, we examine in further detail the anatomical, functional, and molecular mechanisms underlying AUNA1, confirming that diap3-overexpressing Tg mice mimic the human AUNA1 phenotype. Molecular dissection of the apical side revealed that the cytoskeleton meshwork undergoes an aberrant remodeling into the cuticular plate of Tg IHCs at early stages. Strikingly, the overexpression of diap3 leads to an accumulation of microtubules within the IHC cuticular plate. Ultimately, the invasion of microtubules at the apical side of IHCs may interfere with the capability of these sensory cells to transduce incoming acoustic cues.

## Material and Methods

Experiments were carried out in accordance with animal welfare guidelines 2010/63/EC of the European Communities Council Directive regarding the care and use of animals for experimental procedures. Animals were housed in facilities accredited by the French “Ministère de l’Agriculture et de la Forêt” (agreement C-34-172-36), and the experimental protocol was approved (Authorization CEEA-LR- 12111) by the Animal Ethics Committee of Languedoc-Roussillon (France).

### Animals

We studied Tg *diap3*-overexpressing transgenic mice of either sex, which have been previously described (Schoen et al., 2013). In brief, these animals harbor a random insertion of the transgene, composed of the mouse *diap3* gene driven by cytomegalovirus promoter, in the genomic DNA (Schoen et al., 2013). Two lines of mice were obtained, differing in the number of inserted copies of the transgene: eight for line 771 and six for line 924 (Schoen et al., 2013). Mice were bred in-house and maintained on a FVB/NJ genetic background. Line 924 (FVB-Tg(CAG-Diap3)924/Lesp/J is available from Jackson Laboratory (JAX Stock No. 017881). Line 771 (FVB/N-Tg(CAG-Diap3)771Lesp/Mmmh) is available from the Mutant Mouse Research & Resource Center.

### Plasmids

Plasmids used for *diap1-GFP* and *diap3-GFP* expression in HEK293 cells were pReceiver-M03-Diap1 (GeneCopoeia) and pEFmEGFP-mDia2 (Addgene), respectively (GenBank accession numbers: diap1, BC070412.1; diap3, AF094519.1). *Diap2* coding sequence was subcloned from the donor plasmid pCMV6-Ac-GFP-diap2 (Origene) into the receiver plasmid pEGFP-C3 (Takara), using In-Fusion HD Cloning Kit (Takara; National Center for Biotechnology Information Reference Sequence for *diap2*, NM_172493.2). *Diap2* coding sequence was amplified from the donor plasmid using the primers diap2-PEGFP-3-F (5′-GTACTCAGATCTCGAGATGGAGGAGCTCGGGG-3′) and diap2-PEGFP-2-R (5′-TAGATCCGGTGGATCCTTGGATGACATGGCTCCATTG-3′; Eurogentec). The receiver plasmid was digested with XhoI and BamHI restriction enzymes (Promega). Subcloning was performed following the instructions from the manufacturer (Takara). Sequencing of the obtained plasmids for verification was performed by Genewiz.

### Cell culture

HEK293 cell line was maintained in DMEM/F12 culture medium (Invitrogen) with 10% of fetal bovine serum (Gibco) and 1% penicillin/streptomycin (Sigma-Aldrich) at 37°C in a humidified atmosphere under 5% CO_2_.

### Cell transfection and protein extraction

Cells were grown to 60–80% confluence and transfected with plasmids using Lipofectamine 2000 reagent (Invitrogen), following the manufacturer’s instructions. Forty-eight hours after transfection, HEK293 cells were lysed with a lysis buffer (pH 7.6) containing (in mm): 20 Tris-HCl, 100 NaCl, 5 EDTA, 1% Triton X-100, 1 phenylmethylsulfonyl fluoride, and cOmplete Protease Inhibitor Cocktail 1× (Sigma-Aldrich). Samples were placed on an orbital shaker for 2 h at 4°C then centrifuged during 20 min at 13,200 rpm and 4°C. Supernatants were collected, and protein concentration was determined using Pierce BCA Protein Assay Kit (Thermo Fisher Scientific).

### Western blot

Each sample was prepared by mixing 20 µg of protein with loading buffer 1× (Bio-Rad) and 0.05% β-mercaptoethanol (Sigma-Aldrich). Samples were heated for 5 min at 95°C to allow denaturation of the proteins by β-mercaptoethanol. Samples were dropped on 10% polyacrylamide gel (Bio-Rad). After migration, proteins were transferred on membrane (Bio-Rad). Membranes were incubated for 1 h in a blocking buffer containing Tris-buffered saline (TBS) 1×, 0.001% Tween-20, and 3% milk. After blocking, primary antibodies were added into the blocking buffer, and membranes were incubated overnight at 4°C. The used primary antibodies and their respective dilutions were GFP 1:10,000 (Abcam), Diap3 1:20,000 (Eurogentec), and α-tubulin 1:5000 (Abcam). Membranes were washed five times for 5 min with washing buffer containing TBS and 0.001% Tween-20, incubated for 2 h in the blocking buffer containing secondary antibodies conjugated to horseradish peroxidase, and washed five times for 5 min with the washing buffer. After the last washing, membranes were incubated for 1–5 min in ECL solution (Bio-Rad), and staining was revealed using a ChemiDoc MP System instrument (Bio-Rad).

### Genotyping

Transgenic mice were identified by PCR analysis of genomic DNA using FastStart PCR Master Mix (Roche Applied Science). Tail or toe biopsies were digested overnight at 55°C in 300 µl of lysis buffer containing (in mm): 100 Tris-HCL, pH 8.5; 5 EDTA; 0.2% SDS; and 200 NaCl, pH: 8.5, with 100 µg/ml of proteinase K (Promega). Samples were centrifuged for 5 min at 10,000 × *g*, and supernatants were collected. DNA was precipitated by addition of 500 µl of isopropanol. Samples were centrifuged for 10 min at 10,000 × *g*, and supernatants were discarded. DNA was washed with 500 µl of EtOH 70% and centrifuged for 5 min at 10,000 × *g*. After evaporation of the EtOH, DNA was suspended in 100 µl of water. PCR was conducted with the following thermal cycle program: 1 cycle of 95°C for 10 min; 40 cycles of 94°C for 40 s, 62°C for 30 s, and 72°C for 1 min; and a final elongation step at 72°C for 7 min. A part of the exogenous promoter, unique to the transgene and therefore not present in the wild-type (WT) mouse genomic DNA, was detected using the 5′-TGG TTA TTG TGC TGT CTC ATC A-3′ forward primer and the 5′-TTG TCC AGC ATA TCA TCT GTC A- 3′ reverse primer (Eurogentec). Thus, a 300-bp amplicon was obtained only from DNA of transgenic mice, which was visible on a 2% agarose gel electrophoresis of PCR product.

### *In vivo* recordings

Mice were anesthetized by an intraperitoneal injection of a mixture of Zoletil 50 (40 mg/kg) and Rompun 2% (3 mg/kg). Rectal temperature was measured with a thermistor probe and maintained at 37.1°C ± 1°C using a heated underblanket (Homeothermic Blanket Systems, Harvard Apparatus). Heart rate was monitored via electrocardiography.

### Auditory brainstem response and distortion product otoacoustic emission recordings

For ABR, the acoustical stimuli consisted of 9-ms tone bursts, with a plateau and a 1-ms rise/fall time, delivered at a rate of 11/s with alternate polarity by a JBL 2426H loudspeaker in a calibrated free field. Stimuli were presented to the left ear by varying intensities from 100 to 0 dB SPL, in 5-dB steps. Stimuli were generated and data acquired using Matlab (MathWorks) and LabView (National Instruments) software. The difference potential between vertex and mastoid intradermal needles was amplified (2500 times, VIP-20 amplifier), sampled (at a rate of 50 kHz), filtered (bandwidth of 0.3–3 kHz), and averaged (100 to 700 times). Data were displayed using LabView software and stored on a computer (Dell T7400). ABR thresholds were defined as the lowest sound intensity that elicits a clearly distinguishable response. For distortion product otoacoustic emission (DPOAE) recordings, an ER-10C S/N 2528 probe (Etymotic Research), consisting of two emitters and one microphone, was inserted in the left external auditory canal. Stimuli were two equilevel (65 dB SPL) primary tones, f1 and f2, with a constant f2/f1 ratio of 1.2. The distortion 2f1-f2 was extracted from the ear canal sound pressure and processed by HearID auditory diagnostic system (Mimosa Acoustic) on a computer (Hewlett Packard). The probe was self-calibrated for the two stimulating tones before each recording. f1 and f2 were presented simultaneously, sweeping f2 from 20 to 2 kHz by quarter-octave steps. For each frequency, the distortion product 2f1-f2 and the neighboring noise amplitude levels were measured and expressed as a function of f2.

### Electrocochleography

A retroauricular incision of the skin was performed on anesthetized mice, and the left tympanic bulla was opened. Cochlear potentials were recorded with a silver positive electrode placed on the round window membrane. The acoustical stimuli were identical to those used to elicit ABRs except for the alternate polarity. Gross cochlear potentials were amplified (2500 times, VIP-20 amplifier), sampled (at a rate of 50 kHz), filtered (bandwidth of 0.001–20 kHz), averaged (50 to 300 times), displayed with LabView software, and stored on a computer (Dell T7400). The signal was then digitally filtered using Matlab software with a low-pass filter (cutoff frequency 3.5 kHz) to measure the compound action potential and the summating potential, and with a bandpass filter centered on the frequency of stimulation with a 4-kHz span to measure the cochlear microphonic.

### Patch-clamp recordings

After cervical dislocation of mice (postnatal day 13 [P13] to P19), IHCs of the apical coil of freshly dissected organs of Corti were patch-clamped at their basolateral face at room temperature in tight whole-cell or perforated-patch configurations (Nouvian, 2007). The dissection solution contained the following (in mm): 5.36 KCl, 141.7 NaCl, 1 MgCl_2_-6H_2_O, 0.5 MgSO_4_-7H_2_O, 10 HEPES, and 10 d-glucose. For recording of K^+^ currents, the extracellular solution contained the following (in mm): 5.8 KCl, 144 NaCl, 0.9 MgCl_2_-6H_2_O, 1.3 CaCl_2_, 10 HEPES, and 10 d-glucose. The pipette solution for recording of K^+^ currents contained the following (in mm): 135 KCl, 1 MgCl_2_-6H_2_O, 10 HEPES, 2 Mg-ATP, 0.3 Na-GTP, and 5 EGTA. For Ca^2+^ current and capacitance measurement recordings, the extracellular solution contained the following (in mm): 2.8 KCl, 105 NaCl, 1 MgCl_2_-6H_2_O, 2 CaCl_2_, 10 HEPES, 35 TEA-Cl, 1 CsCl, and 10 d-glucose. The pipette solution for whole-cell recordings of Ca^2+^ currents contained the following (in mm): 135 Cs-glutamic acid, TEA-Cl, 10 4-AP, 1 MgCl_2_-6H_2_O, 10 HEPES, 2 Mg-ATP, and 0.3 Na-GTP. The pipette solution for perforated patch recordings contained the following (in mm): 135 KCl, 10 HEPES, 1 MgCl_2_, and 400 μg/ml amphotericin B. Solutions were adjusted to pH 7.3 and had osmolarities between 290 and 310 mOsm/kg H_2_O. All chemicals were obtained from Sigma-Aldrich, with the exception of amphotericin B (Calbiochem). Patch pipettes were pulled from borosilicate glass capillaries (Kwik Fil, WPI) with a two-step vertical puller PIP 6 (HEKA Elektronik) and coated with silicone elastomer (Sylgard).

### K^+^ current recordings and resting membrane potentials

Currents were low-pass filtered at 5 kHz and sampled at 50 kHz. Series resistance (Rs) was compensated online (80%; *t* = 10 μs), and all voltages were corrected for the voltage drop across the uncompensated series resistance (7.25 ± 0.31 MOhm for WT mice, 9.12 ± 0.99 MOhm for Tg mice) and for liquid junction potentials (–4 mV) measured between intra- and extracellular solutions. Except for the deactivated current recordings (KCNQ conductances), all currents were leak-corrected using P/n protocol (10 leak pulses with amplitudes of 20% of the original pulse from a holding potential of –104 mV).

### Ca^2+^ current recordings

Currents were low-pass filtered at 5 kHz and sampled at 10 kHz for exocytic cell membrane capacitance change (ΔCm) and at 40 kHz for Ca^2+^ current recordings. Ca^2+^ current was isolated using P/n protocols (10 leak pulses with amplitudes of 20% of the original pulse from a holding potential of –117 mV). Ca^2+^ current integrals were calculated from the total depolarization-evoked inward current, including Ca^2+^ tail currents after P/n leak subtraction (i.e., from the start of the depolarization step to 1.5 ms after the end of the depolarization step). Cells that displayed a membrane current exceeding –50 pA at –87 mV were discarded from the analysis. No Rs compensation was applied, but recordings with Rs >30 MOhm and >15 MOhm for perforated and whole-cell patch-clamp experiments, respectively, were discarded from the analysis. All voltages were corrected for liquid junction potentials calculated between pipette and bath (–17 mV).

### Capacitance measurement recordings

Cell membrane capacitance (Cm) was measured using the Lindau–Neher technique (Lindau and Neher, 1988), implemented in the software-lockin module of Patchmaster (HEKA Elektronik) combined with compensation of pipette and resting cell capacitances by the EPC-10 (HEKA Elektronik) compensation circuitries. A 1 kHz, a 70-mV peak-to-peak sinusoid was applied about the holding potential of –87 mV. ΔCm was estimated as the difference of the mean Cm over 400 ms after the end of the depolarization (the initial 250 ms was skipped), and the mean prepulse capacitance (400 ms). Mean ΔCm estimates present grand averages calculated from the mean estimates of individual IHCs.

### Immunohistochemistry and confocal microscopy

Immunohistochemistry was performed on whole-mount preparations of organs of Corti. The mice were decapitated under deep anesthesia using 50 mg/kg pentobarbital, and their cochleas were removed from the temporal bone and dissected in the patch-clamp dissecting solution. The apical turns of the cochleas were then fixed for 15 min in 4% paraformaldehyde diluted in phosphate buffer (0.1 M, pH 7.3, 4°C); afterward, they were immunohistochemically processed as a whole-mount. The tissues were rinsed three times for 10 min in PBS containing (in mm) 130 NaCl, 2.68 KCl, 10 Na_2_HPO_4_, and 1.47 KH_2_PO_4_, preincubated for 30 min in blocking solution (10% donkey serum, 0.3% Triton X-100), then incubated overnight at 4°C in the incubation solution (1% donkey serum, 0.1% Triton X-100) with primary antibodies or antisera. The primary antibodies used and their respective dilutions were CtBP2 1:1000 (BD Transduction Laboratories; RRID: AB_399431), GluA2/3 1:50 (RRID: AB_90710), spectrin 1:400 (EMD Millipore; RRID AB_11214057), α1 tubulin 1:500 (RRID: AB_521686), β1 tubulin 1:10 and β2 tubulin 1:500 (Novus Biologicals; RRID: AB_792489), β4 tubulin 1:200 (RRID:AB_297919), and acetylated α-tubulin 1:500 and detyrosinated α tubulin 1:500 (Abcam; RRID:AB_869990). The diap3 antiserum was produced by Eurogentec. It was raised in guinea pig against a specific motif of mouse diap3 (amino acids 94–105: LSSETMENNPKA). The dilution used was 1:200. Tissues were then rinsed three times for 10 min in wash buffer containing (in mm) 15.8 Na_2_HPO_4_, 2.9 NaH_2_PO_4_, 0.3% Triton X-100, and 450 NaCl and incubated for 2 h in the incubation solution with fluorescently labeled secondary antibodies, rhodamine-phalloidin (Molecular Probes) and/or Hoechst dye (Invitrogen). They were finally rinsed three times for 10 min in wash buffer and mounted in Prolong Gold Antifade Reagent (Fisher Scientific). Tissues were examined with the Zeiss LSM780 or Leica SP8-UV confocal microscopes of the Montpellier RIO Imaging facility (Montpellier, France). Image stacks were then processed with ImageJ software (National Institutes of Health). For fluorescence analysis and synapse counting, image stacks were processed with Matlab software. The juxtaposed spots of the presynaptic ribbon component RIBEYE and postsynaptic glutamate receptors subunits GluA2/3 allowed the quantification of the ribbon-containing synapses per IHC. For each condition (antibody, age, genotype), at least three cochleas were examined.

### Electron microscopy

Scanning (SEM) and transmission (TEM) electron microscopy were done for morphologic evaluation of the cochlear hair cells. For both techniques, the animals were decapitated under deep anesthesia (pentobarbital, 50 mg/kg), and cochleas were removed from the temporal bone, perfused with a solution of 2.5% glutaraldehyde in 0.1 m phosphate buffer (pH 7.3–7.4), and immersed in the same fixative for 1 h at room temperature. For SEM, the bony capsule of the cochlea was dissected out, and the stria vascularis as well as the tectorial and Reissner’s membranes were removed. After being rinsed in phosphate buffer, the samples were dehydrated in a graded series of ethanol (30–100%), critical-point dried in CO_2_, coated with gold–palladium, and observed under a Hitachi S4000 microscope. Examinations were made all along the cochlear spiral (∼6 mm long) from the apex to the basal end. For each condition (age and genotype), at least three cochleas were processed and examined using SEM. For TEM, the cochleas were postfixed in a 2% aqueous solution of osmium tetroxide for 1 h, rinsed in phosphate buffer, dehydrated in a graded series of ethanol (30–100%), and embedded in Epon resin. Ultrathin radial sections of the organ of Corti were cut in the 16-kHz region of the cochlea, mounted on formvar-coated grids, stained with uranyl acetate and lead citrate, and observed using a Hitachi 7100 microscope. For TEM, we examined one or two cochleas at each age in the Tg 924 line.

### Data analysis

Data were analyzed using Matlab and Igor Pro (WaveMetrics) software. All data are expressed as mean ± SEM and were compared by Student *t*-test and Wilcoxon test.

## Results

### Diap3-overexpressing mutant mice replicate AUNA1

**Figure 1.**
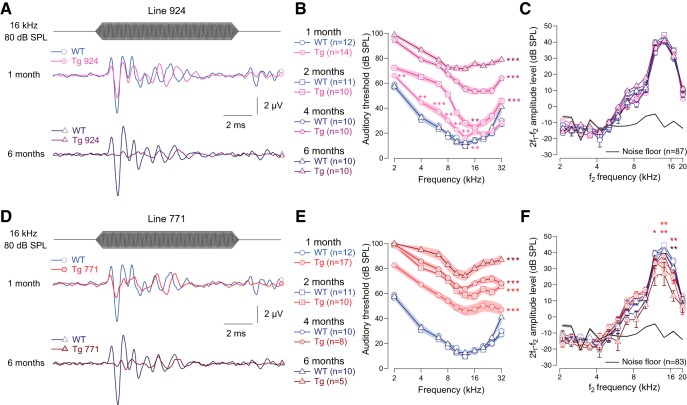
Transgenic mouse lines 924 (Tg 924) and 771 (Tg 771) mimic human AUNA1 deafness. ***A***, ***D***, Representative ABR recordings evoked by 16-kHz tone burst at 80 dB SPL from 1- and 6-month-old WT, Tg 924 (***A***), and Tg 771 (***D***) mice. ***B***, ***E***, Mean ABR audiograms from 1- to 6-month-old WT, Tg 924 (***B***), and Tg 771 (***E***) mice. ***C***, ***F***, DPOAEs from 1- to 6-month-old WT, Tg 924 (***C***), and Tg 771 (***F***) mice. The mean 2f1-f2 amplitude level is shown as a function of f2 frequency. The black line indicates the background noise level. *n* indicates the number of cochleae recorded. Level of significance: *p < 0.05; **p < 0.01; ***p < 0.001 (Wilcoxon test). For ***B*** and ***E***, asterisks located next to the audiograms indicate a similar *p*-value for all the frequencies, except when otherwise noted.

We examined the auditory system of two lines of Tg mice overexpressing the diap3 protein (Tg 771 and Tg 924). In response to incoming sound stimulation, both Tg lines showed a progressive reduction in ABR amplitude (Fig. 1*A*, *D*). In addition, both Tg lines showed a progressive threshold shift (Fig. 1*B*, *E*). Hearing impairment progressed more rapidly in the Tg 771 mice compared with the Tg 924 mice. Despite a slight reduction in the DPOAE amplitude in Tg 771 mice, both lines showed robust DPOAEs, indicating that OHC activity was essentially preserved (Fig. 1*C*, *F*). Altogether, the two Tg lines replicate the hallmarks of AUNA1 deafness, i.e., progressive hearing loss together with functional cochlear amplification.

### Altered transduction in IHCs of diap3-overexpressing mutant mice

**Figure 2.**
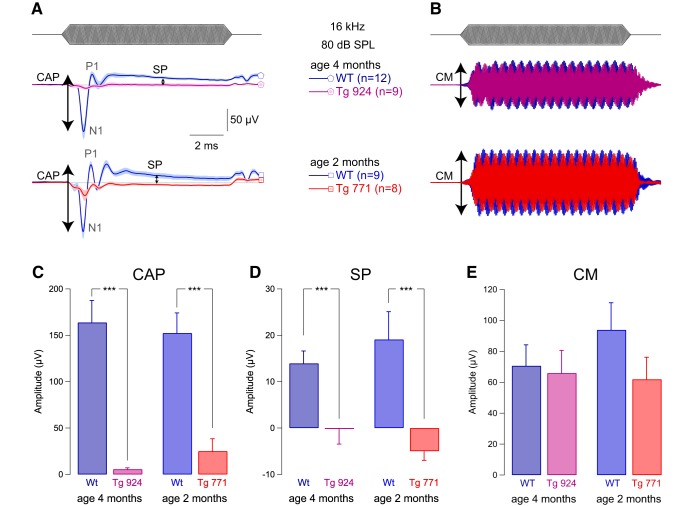
IHC receptor potential is altered in diap3-overexpressing mice. ***A***, Mean compound action potential (CAP; N_1_-P_1_ amplitude) and summating potential (SP), reflecting the auditory afferent fiber activation and IHC receptor potential, respectively. ***B***, Cochlear microphonic (CM), reflecting the OHC activation. CAP, SP, and CM were evoked by 16-kHz tone burst at 80 dB SPL in 4-month-old WT and Tg 924 mice and 2-month-old WT and Tg 771 mice. ***C–E***, Mean CAP amplitude (***C***), SP amplitude (***D***), and CM amplitude (***E***) from 4-month-old WT and Tg 924 mice and 2-month-old WT and Tg 771 mice. *n* indicates the number of cochleae recorded. Level of significance: ***p < 0.001 (Wilcoxon test).

To better understand the mechanisms underlying AUNA1, we measured the activity of the sensory hair cells *in vivo* using electrocochleography. Compound action potential was strongly diminished in both Tg lines (Fig. 2*A*, *C*). In addition, we found a drastic reduction in the summating potential amplitude (Fig. 2*A*, *D*). Consistent with robust DPOAEs in the Tg mice, both WT and Tg lines showed comparable strong cochlear microphonic responses (Fig. 2*B*, *E*). These results illustrate a selective alteration of IHC function in the Tg mice. Moreover, the reversal behavior of the summating potential, i.e., negative amplitude in the diap3-overexpressing mice, is reminiscent of changes in the ionic conductances of IHCs (Bobbin et al., 1990, 1991) or in the resting position of the stereocilia displacement-response curve (Corey and Hudspeth, 1983; Farris et al., 2006; Johnson et al., 2011).

### Normal synaptic vesicle recycling in diap3-overexpressing mutant mice

**Figure 3.**
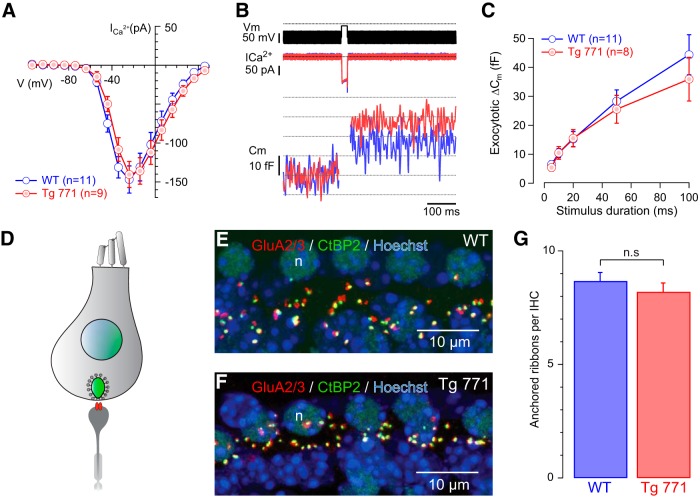
Ca^2+^-triggered exocytosis is not impaired in diap3-overexpressing mice. ***A***, Ca^2+^ current steady-state I/V relationships of WT (blue) and Tg 771 (red) mouse IHCs P13–P16. Steady-state amplitude was measured as the average over the last 5 ms of the 10-ms test pulse. ***B***, Ca^2+^ current (ICa^2+^) and Cm traces, low-pass filtered at 100 Hz (from top to bottom) of representative WT (blue) and Tg (red) IHCs elicited by 20-ms depolarization to the peak Ca^2+^ current potential. ***C***, Kinetics of exocytosis (Cm) of WT (blue) and Tg (red) IHCs. ***D***, Scheme illustrating the synapse between IHC and afferent neuron. The IHC nucleus is shown in blue, the synaptic ribbon in green, and the postsynaptic glutamate receptor in red. ***E–G***, Comparable number of IHC ribbon synapses between WT and Tg 771 mice. Presynaptic ribbons and postsynaptic glutamate receptors are labeled using antibodies against CtBP2 and GluA2/3, respectively (***E***, ***F***). ***G***, Note the comparable numbers of ribbon-containing afferent synapses per IHC from 1-month-old WT mice (8.67 ± 0.38 synapses/IHC, *n* = 79 IHCs examined) and Tg 771 mice (8.19 ± 0.39, *n* = 92 IHCs examined). No significant difference was found (*p* > 0.05), n.s: not significant.

To determine whether the stimulation-secretion coupling is affected by the diap3 overexpression, calcium-triggered exocytosis was probed using patch-clamp recordings (Fuchs et al., 2003). Because the Tg 771 line already showed a threshold shift occurring immediately after the onset of hearing (threshold: 16.1 ± 1.4 dB SPL and 28.3± 1.7 dB SPL in P19-P22 WT and Tg 771, respectively, in response to 10-kHz tone burst; WT, *n* = 9; Tg 771, *n* = 3; *p <* 0.05, Wilcoxon test), membrane capacitance, reflecting synaptic vesicle exocytosis, was measured in IHC from 2 to 4 weeks of age in this Tg line (Fig. 3). No difference was observed in the amplitude or in the voltage activation of the calcium current between WT and Tg 771 mice (Fig. 3*A*). In addition, membrane capacitance jumps evoked by calcium influx of different durations were comparable between WT and Tg 771 mice (Fig. 3*B–C*). Furthermore, the number of ribbon synapses was not altered in 1-month-old Tg 771 mice (Fig. 3*D–G*). Taken together, these data exclude a primary defect at the IHC ribbon synapse in AUNA1.

### Potassium currents are not affected in diap3-overexpressing mutant mice

**Figure 4.**
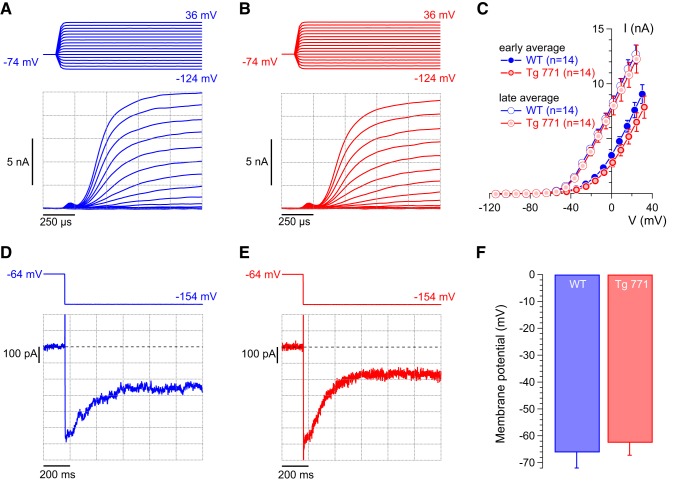
Normal potassium currents in diap3-overexpressing mice. ***A***, ***B***, Representative outward currents recorded from 1-month-old WT (blue) and Tg 771 (red) IHCs. Currents were evoked by step depolarizations from a holding potential of –74 mV to the indicated potentials (voltage increment of 10 mV). ***C***, Average I-V relationships for WT (in blue) and Tg 771 (in red) IHCs derived from averaging currents 375 µs after the start of the depolarizing pulses over 250 µs (early average) and over the last 50 ms of depolarization steps (late average). Early and late averages indicate, respectively, fast- and slow- activating potassium current, corresponding to BK and delayed-rectifier channels. *n* indicates the number of IHCs recorded. ***D***, ***E***, Representative current traces obtained from 1-month-old WT (blue, ***D***) and Tg 771 (red, ***E***) IHCs. Currents were elicited by a voltage step from a holding potential of –64 mV to a hyperpolarized potential of –154 mV. Zero current level is indicated by a dotted line. Note that the current was already activated at the resting potential of approximately –60 mV and became deactivated upon hyperpolarization, which is indicative of KCNQ4 currents. ***F***, Mean resting membrane potential of WT (blue) and Tg 771 (red) IHCs (no current injection). The resting membrane potentials were not significantly different between the genotypes.

Because IHC potassium conductances contribute to the receptor potential (Corey and Hudspeth, 1983), we then recorded the different potassium currents in IHC. In response to depolarizing steps, IHCs elicit fast and slow outward potassium currents, carried by BK and delayed-rectifier channels, respectively (Kros and Crawford, 1990; Marcotti et al., 2003; Oliver et al., 2003). No difference in the amplitude or voltage activation of K^+^ currents was measured between IHCs of WT and Tg 771 mice (Fig. 4*A–C*). In addition, IHCs from Tg 771 mice expressed a deactivating potassium current, which is carried by the KCNQ4 potassium channel and sets the resting membrane potential (Fig. 4*D*, *E*; Marcotti et al., 2003; Oliver et al., 2003). Accordingly, the resting membrane potential in IHCs of Tg 771 mice lies in the same voltage range as IHCs of WT mice (Fig. 4*F*). Therefore, a change in potassium conductance cannot account for the defective receptor potential in AUNA1 deafness.

### Distortion of the IHC cuticular plate is the primary defect in Tg mice

**Figure 5.**
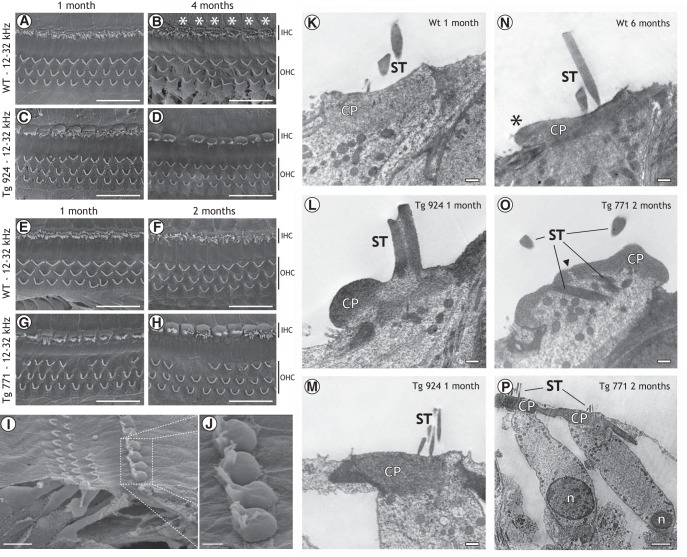
Morphological defects at the cuticular plate and stereociliar bundles in inner hair cells of diap3-overexpressing mice. ***A–J***, SEM of the organ of Corti in 1-month-old (***A***, ***E***), 4-month-old (***B***), and 2-month-old (***F***) WT mice, 1-month-old (***C***) and 4-month-old (***D***, ***I***, ***J***) Tg 924 mice, and 1-month-old (***G***) and 2-month-old (***H***) Tg 771 mice. Note the cuticular plate lump (white asterisk) in WT IHCs starting from 4 months old (***B***). In the Tg lines (***C***, ***D***, ***G–J***), a severe swelling of the IHC cuticular plate is observed for all ages. The alteration of the cuticular plate can be associated with disarrayed or fused stereociliar bundle. In addition, some OHCs are missing. ***K–P***, TEM from hair cells in 1-month-old (***K***) and 6-month-old (***N***) WT mice, 1-month-old Tg 924 mice (***L***, ***M***), and 2-month-old Tg 771 mice (***O***, ***P***). ***K***, In 1-month-old WT mice, the cuticular plate (CP) forms an electron-dense matrix, which is homogeneously distributed at the IHC apical pole, except at the fonticulus. Note the presence of numerous mitochondria below the CP and the well-organized stereocilia (ST) above. ***N***, In 6-month-old WT mice, the CP still has a homogeneous distribution. Note that the CP area expands over the neighboring supporting cell (black asterisk), although TEM did not show swelling comparable to that seen in the Tg mice. ***L***, In 1-month-old Tg 924 mice, the CP dramatically accumulated at the border of the IHC and the ST are fused. ***M***, In 1-month-old Tg 924 mice, the apical pole of OHCs looks normal, with its CP anchoring the ST bundle. ***O***, In 2-month-old Tg 771 mice, the ST are embedded in the IHC cytoplasm. The CP is not confined to its regular position, and the apical pole of the IHC protrudes toward the scala media. Note the discontinuous CP (arrow). ***P***, In 2-month-old Tg 771 mice, OHCs show a normal appearance, with the CP anchoring the ST bundle. Scale bars: ***A–H***: 20 µm, ***I***: 10 µm, ***J***: 2.5 µm, ***K–O***: 500 nm, ***P***: 2 µm.

**Figure 6.**
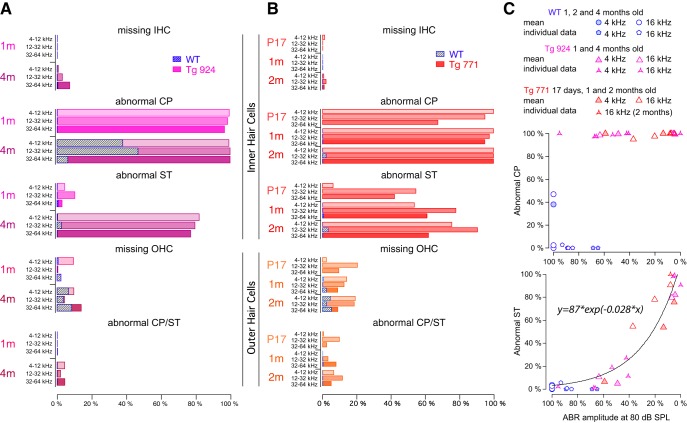
Quantification of the anatomical alteration in hair cells of diap3-overexpressing mice. ***A***, ***B***, Abnormal CP indicates the increase of the cuticular plate area. Abnormal ST indicates disarrayed or fused stereocilia. Note that 4-month-old WT mice exhibit a large cuticular plate that could not be easily distinguished from diap3-overexpressing mice. For OHCs, abnormal CP and ST were pooled because of the minimal defect observed in these cells. ***C***, Top, fraction of IHCs with an abnormal CP plotted against the reduction in the ABR amplitude probed at 80 dB SPL. ABR with various amplitude reductions (0–90%) can be measured with 100% IHCs harboring a swollen CP. ***C***, Bottom, fraction of IHCs harboring an abnormal ST plotted against the reduction in the ABR amplitude probed at 80 dB SPL. The relationship between the reduction in ABR amplitude and the alteration in the stereociliar bundle can be fitted by the following exponential: *y* = *a* × exp(*b* × *x*), where *y* is the fraction of IHCs with abnormal stereocilia, *x* is the reduction in the ABR amplitude, and *a* and *b* are constants. For ***C***, the mean values correspond to the degree of anatomical damage in the 4- and 16-kHz coding frequency regions from 1-, 2-, and 4-month-old mice and plotted against the ABR amplitude average at the corresponding coding frequencies (4 and 16 kHz) from different groups of 1-, 2-, and 4-month-old mice. Individual data values correspond to the anatomofunctional correlation that has been obtained in the same mice.

As the lack of receptor potential can be explained by a damage of the mechanotransducer apparatus (Corey and Hudspeth, 1983; Farris et al., 2006), the morphology of the hair cell apical pole was assessed using electron microscopy. In WT mice, stereocilia were nicely organized and anchored into the cuticular plate, which showed a normal appearance (Fig. 5*A*, *E*, *F*, *J*). As early as age 4 months, the WT IHC cuticular plate showed a little knob (Fig. 5*B*, *M*). In contrast, the diap3-overexpressing Tg mice showed a severe alteration at the IHC apical side (Fig. 5*C*, *D*, *G–I*, *K*, *N*). In both Tg lines, the cuticular plates of IHCs were drastically swollen all along the cochlea (Fig. 5*C*, *D*, *G–I*). Using TEM, the cuticular plate seemed to be pushed toward the IHC periphery opposite to the stereociliary bundle, leading to its accumulation at the upper side of the cell (Fig. 5*K*). The displacement of the cuticular plate could be also associated with disarrayed or fused stereocilia (Fig. 5*K*). Integration of fused stereocilia into the cell cytoplasm was also observed (Fig. 5*N*). Interestingly, the cuticular plate distortion started promptly after the onset of hearing, whereas changes in the stereocilia organization progressively increased over the lifespan of the mice (Fig. 6*A*, *B*). In contrast, OHCs from diap3-overexpressing mice retained a normal morphology (Fig. 5*C*, *D*, *G–I*, *L–O*), although a few of them harbored a distorted apical pole (abnormal cuticular plate and stereociliary bundle) or were missing (Fig. 5*G–I*).

To differentiate between the IHC cuticular plate swelling and the distorted stereocilia as the cause of the hearing loss, we correlated the morphological features to the ABR at different frequencies and ages (Fig. 6*C*). Plots of anatomical defect scores against the ABR amplitude demonstrated that hearing loss was independent of the cuticular plate distortion (Fig. 6*C*, top) but was highly correlated with fused stereocilia (Fig. 6*C*, bottom). Of note, the number of IHCs harboring a distorted cuticular plate in the 4-month-old WT mice is likely to be overestimated, since our 2D SEM screen could not discriminate the cuticular plate lump in the 4-month-old WT mice from the swelling in the diap3-overexpressing mice at this age. Nevertheless, these results suggest that (1) the IHC cuticular plate abnormalities precede the defects of the stereociliary bundle, and (2) hearing loss ultimately develops due to the disorganization of stereocilia, which most likely impedes the activity of the mechanotransducer channel.

### Molecular correlates of cuticular plate remodeling

**Figure 7.**
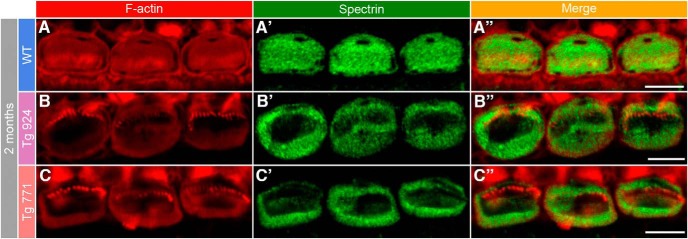
Disorganization of the cuticular plate components. ***A–A″***, In 2-month-old WT IHCs, F-actin (red, ***A***) and spectrin (green, ***A′***) are homogeneously distributed in the whole cuticular plate, except for the kinocilium imprint. ***A″***, Merge of both labels. ***B–C″***, In 2-month-old Tg 924 (***B–B″***) and Tg 771 (***C–C″***) mice, actin filaments and spectrin are concentrated toward the periphery of the IHC apical part, leaving an empty space at the cuticular plate center. Scale bars: 5 µm.

The morphological distortion of the cuticular plate was then examined at the molecular level. Phalloidin-rhodamine and spectrin immunostainings indicate a severe alteration in the shape of the cuticular plate. While the F-actin as well as spectrin networks appear homogenously distributed in the cuticular plate of WT IHCs, they tend to concentrate at the border of IHCs from both Tg lines, leaving F-actin- and spectrin-free spots in the middle of the cuticular plate (Fig. 7). In contrast, the F-actin and spectrin distribution were not affected in the cuticular plate of OHCs (data not shown). Thus, the drastic remodeling of the cuticular plate molecular components may account for the anatomical alteration observed in electron microscopy.

### Aberrant microtubule meshwork distribution in Tg mice

**Figure 8.**
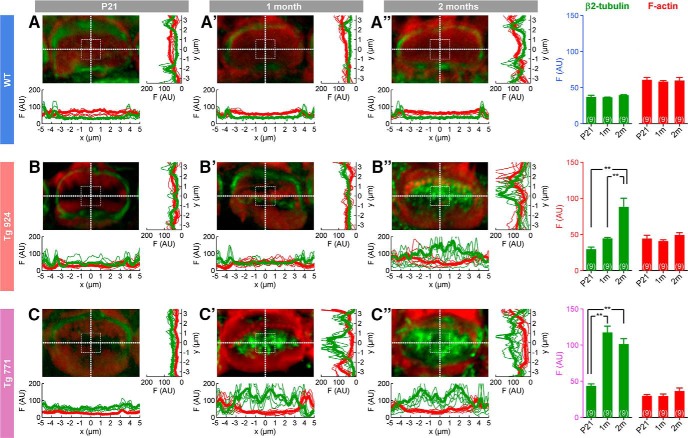
Microtubule network remodeling in IHCs of diap3-overexpressing mice. ***A–C″***, Fluorescence intensity distribution of microtubules (green) and F-actin (red) at the cuticular plate section through *x* and *y* sections in WT (***A–A″***), Tg 771 (***B–B″***) and Tg 924 (***C–C″***) lines at P21 (***A***, ***B***, ***C***), 1 month (***A′***, ***B′***, ***C′***), and 2 months (***A″***, ***B″***, ***C″***). Actin filaments are labeled using phalloidin-rhodamine (red), and microtubules are stained using an antibody against the β2-tubulin subunit (green). Pictures show a high magnification of the cuticular plate section over a single IHC. Thick and thin lines represent the average and individual fluorescence intensities (*n* = 9 IHCs in each condition), respectively. Right, histograms show the fluorescence average from an area of 4 µm^2^ located at the image center for the different genotypes and age. AU, arbitrary unit. **p < 0.01 (Student’s *t*-test).

**Figure 9.**
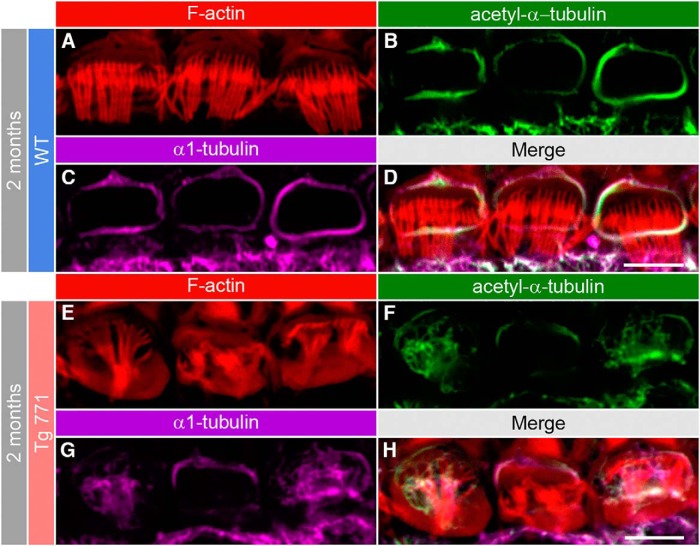
Acetylated microtubules populate the cuticular plate center in the diap3-overexpressing mice. ***A–H***, F-actin and microtubule distribution at the IHC apical side from 2-month-old WT (***A–D***) and Tg 771 (***E–H***) mice. Actin filaments are labeled by phalloidin-rhodamine (red, ***A***, ***E***), and microtubules are stained using antibody against acetyl-α1-tubulin (green, ***B***, ***F***) or against the α1-tubulin subunit (magenta, ***C***, ***G***). Scale bars: 5 µm.

Next, we sought to decipher the mechanism governing the cuticular plate defect in Tg IHCs. Because of the displacement of the cuticular plate, we reasoned that molecular scaffolding proteins interacting with the cuticular plate might be mistargeted or disrupted. In this framework, microtubules are potential candidates of interest, as they are known to surround the cuticular plate (Steyger et al., 1989; Furness et al., 1990; Jensen-Smith et al., 2003) and to be downstream targets of the diaphanous family members (Ishizaki et al., 2001; Palazzo et al., 2001; Wen et al., 2004; Bartolini et al., 2008). In WT IHCs, β2-tubulin subunit immunostainings revealed a ring-like distribution of microtubules around the cuticular plate (Fig. 8*A–A″* ). In the Tg 771 and Tg 924 lines, microtubules were found inside the cuticular plate center (Fig. 8*B*, *C*). α1-, β1-, and β4-tubulin subunits showed a similar pattern of distribution, i.e, inside the IHC cuticular plate in the diap3-overexpressing mice (data not shown). Close examination showed that microtubules preferentially populated the F-actin–free spot within the cuticular plate. At 1 month, Tg 771 IHCs already showed a large immunolabeling of microtubules within the cuticular plate center, whereas Tg 924 IHCs displayed a more progressive distribution of microtubules (Fig. 8*B*, *C*). At later stages, the immunostaining intensity of microtubules ring tended to be reduced in the Tg mice, suggesting a loss of the tubulin subunits that surround the cuticular plate (Fig. 8*B*, *C*). The strong overlap between the immunolabels with antibodies against the α1 and its acetylated form suggests that the aberrant microtubules within the cuticular plate were stable (Fig. 9). The distribution of microtubules was not changed in OHCs (data not shown). These results suggest that in both Tg mice, aberrant distribution of microtubules may exert mechanical constraints against the cuticular plate and thus be responsible for the primary defect in IHCs.

### Early onset of microtubule meshwork remodeling

**Figure 10.**
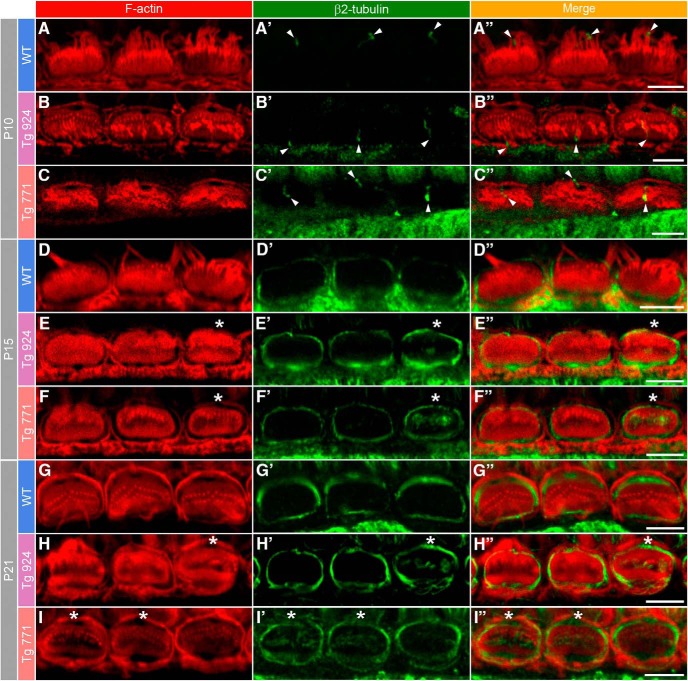
Early onset of the microtubule remodeling. F-actin and microtubule distribution at the IHC apical side from WT (***A***, ***D***, ***G***), Tg 924 (***B***, ***E***, ***H***), and Tg 771 (***C***, ***F***, ***I***) mice at P10, P15, and P21. Actin filaments are labeled by phalloidin-rhodamine (red), and microtubules are stained using antibody against the β2 tubulin subunit (green). ***A–C″***, At P10, the β2-tubulin staining is reminiscent of the kinocilium position (arrowheads). In 2- and 3-week-old WT mice, microtubules are distributed around the cuticular plate (green, ***D–D″***, ***G–G″***). In the P15 Tg 924 (***E–E″*** and ***H–H″***), and P21 Tg 771 (***F–F″*** and ***I–I″***), microtubules can be readily distinguished in the center of the cuticular plate below the stereociliar bundle (asterisk). Scale bars: 5 µm.

If the microtubule rearrangement is responsible to some extent for the cuticular plate distortion, which is observed right after the onset of hearing, we should then expect an early onset of abnormal distribution of the microtubules within the cuticular plate. Before the onset of hearing, tubulin immunostaining was confined to the kinocilium area, but no ringlike distribution was conspicuously observed at this stage (Fig. 10*A–C*). After the onset of hearing, microtubules started to localize around the cuticular plate in the WT mice (Fig. 10*D*, *G*). In Tg 771 and Tg 924 mice at P15 and P21, respectively, we also observed isolated microtubules at the cuticular plate center, below the stereociliary bundle (Fig. 10*E*, *F*, *H*, *I*). At later stages, microtubules were also found invading the stereociliary bundles. Taken together, these results suggest that microtubule meshwork remodeling occurs at early stages, invading the cuticular plate center, and coincides with the morphological swelling of the cuticular plate.

### Diap3 accumulates at the IHC cuticular plate in Tg mice

**Figure 11.**
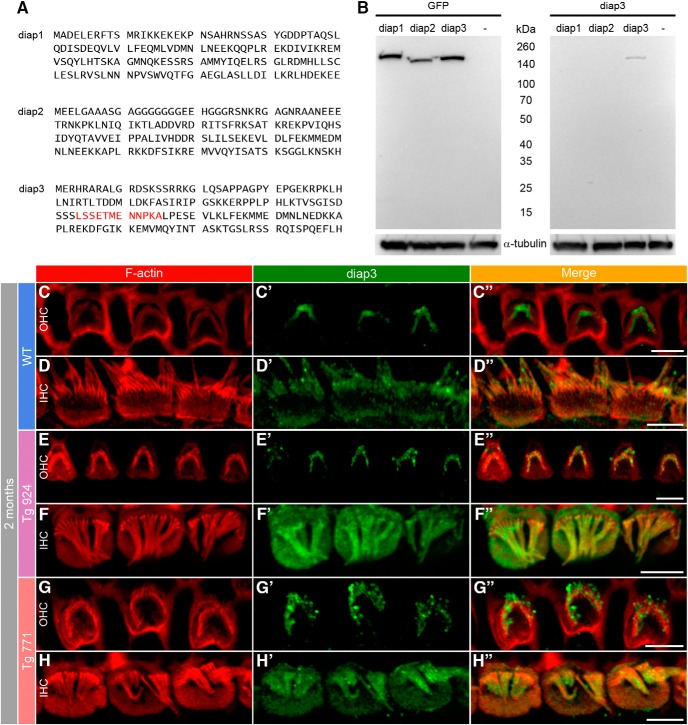
Accumulation of diap3 in the cuticular plate of IHCs of diap3-overexpressing mice. ***A***, Amino acid (1–120) sequence of mouse diap1, diap2, and diap3. The LSSETMENNPKA motif in the diap3 protein sequence (red) has been used to generate the diap3 antibody. ***B***, Western blot analysis of diap3 antibody specificity. HEK293 cells were transfected in parallel with plasmids encoding the GFP-diap1, GFP-diap2, or GFP-diap3 fusion protein. Protein extracts from HEK293 cells were probed with GFP (upper right) and diap3 (upper left) antibodies. –, nontransfected HEK293 cells. GFP staining indicates efficient expression for each diap-GFP fusion protein in HEK293 cells (at ∼160 kDa). Note that the diap3 antibody recognizes diap3-GFP protein but not diap1-GFP or diap2-GFP proteins. On lower panels, α-tubulin staining is used as protein loading control. ***C–D″***, F-actin and diap3 localization at the apical side of OHCs (***C–C″***) and IHCs (***D–D″***) in 2-month-old WT mice. Actin filaments are labeled by phalloidin-rhodamine (red, ***C–D***), and diap3 is stained using an anti-diap3 antibody (green, ***C′–D′***). Diap3 is preferentially localized inside the stereocilia of both hair cells (***C′–D′***) and within the cuticular plate of IHCs (***D′***). ***C″–D″***, Merge of both immunolabels. ***E–H″***, Localization of F-actin and diap3 in 2-month-old Tg 924 (***E–F″***) and Tg 771 (***G–H″***) mice. Actin filaments are concentrated toward the periphery of the IHC apical part, leaving an actin-free zone in the IHC cuticular plate center (***F***, ***H***). In Tg 771 (***E′–F′***) and Tg 924 (***G′–H′***) mice, diap3 accumulates in the cuticular plate and stereocilia of IHCs (***F′–H′***), and only shows a stereociliary bundle localization in OHCs (***E′–G′***). Scale bars: 5 µm.

The massive change in the microtubule distribution may be due to a preferential targeting of diap3 at the apical side of hair cells. To probe this hypothesis, we generated an antibody against diap3 (Fig. 11*A*, *B*). In our assay, the diap3 antibody did not recognize diap1 and diap2, suggesting that this antibody is specific against diap3. Further demonstration for specificity would require the use of a viable diap3-knockout mouse (Watanabe et al., 2013). Nevertheless, using immunohistochemistry, we observed a labeling of the stereocilia in both WT IHCs and OHCs, as well as immunostaining of diap3 in the IHC cuticular plate (Fig. 11*C*, *D*). In the Tg mouse lines, diap3 was located in the IHC cuticular plate and in the stereocilia overlapping the actin distribution (Fig. 11*F–H*). However, we did not find expression of diap3 in the cuticular plate of Tg OHCs (Fig. 11*E–G*). These data suggest that diap3 is a component of the stereociliary bundle in both IHC and OHC and accumulates preferentially at the apical part of IHCs in the Tg lines.

## Discussion

We showed that the overexpression of diap3 in mouse mimics the auditory neuropathy 1 (AUNA1), i.e., the loss of the IHC transduction process without affecting OHC amplification. Neurotransmitter release and potassium currents in IHCs were not altered in the diap3-overexpressing mice. However, we distinguished two different steps in the mutant mice: the distortion of the IHC cuticular plate followed by the loss of stereociliary bundle integrity. The aberrant localization of microtubules at the center of the cuticular plate makes the remodeling of the microtubule network an attractive mechanism accounting for the cuticular plate alteration and the AUNA1 deafness.

### Microtubule meshwork remodeling in diap3-overexpressing mutant mouse

Diap3 belongs to the formin family, which is known to nucleate and elongate actin filaments and to stabilize microtubules (Ishizaki et al., 2001; Palazzo et al., 2001; Pruyne et al., 2002; Wen et al., 2004; Bartolini et al., 2008). In the WT mouse, our results show that diap3 is preferentially expressed in the stereociliary bundle of both hair cells and is also found in the cuticular plate of IHCs. Because of the large abundance of actin at the hair cell apical side (Flock and Cheung, 1977; Slepecky and Chamberlain, 1985; Furness et al., 2005), it is tempting to consider diap3 as an integral component of the transduction apparatus, with a key role in actin turnover (Schneider et al., 2002; Zhang et al., 2012; Drummond et al., 2015). Consistent with the diap3 localization that we demonstrate, a proteomic study has recently identified diap3 in the mouse vestibular hair cell stereociliary bundle (Krey et al., 2015). However, we cannot completely exclude a nonspecific diap3 staining in the stereociliary bundle, because the intensity of staining in the diap3-overexpressing mice is low. Moreover, the molecular remodeling of the cuticular plate cytoskeleton network in the IHC diap3-overexpressing Tg mice is consistent with our observation of the sole diap3 localization within the cuticular plate.

In the diap3-overexpressing Tg mice, diap3 seems to be properly distributed along the hair cell stereociliary bundle and, in addition, colocalizes with actin at the IHC cuticular plate. Thus, the abundant expression of diap3 results in its accumulation at the cuticular plate, which may in turn lead to a larger amount of actin (Ishizaki et al., 2001; Pruyne et al., 2002). In this scenario, the excess actin would expand the cuticular plate and lead to a swollen appearance under electron microscopy. Because of the dense and packed structure of the cuticular plate, the remodeling of the actin network would drag together the other components of the cuticular plate, such as spectrin (Drenckhahn et al., 1991; Furness et al., 2008; Vranceanu et al., 2012). However, under this hypothesis, the increase in actin volume would be homogeneous within the cuticular plate of the hair cells. In contrast, the cuticular plate shows actin- and spectrin-free zones, below the stereociliary bundle, which increased in size over time. We found that microtubules populate the center of the IHCs cuticular plate, i.e., in the actin-free area, of the diap3-overexpressing Tg mice. Thus, the accumulation of diap3 at the cuticular plate may stabilize microtubules at an aberrant place. The excess of microtubules may in turn apply a mechanical force against the cuticular plate, and then displace the cuticular plate toward the periphery of IHCs.

The distribution of microtubules within the cuticular plate center, i.e., in the actin-free zones, could be explained by the invasion of the pool of microtubules that surrounds the cuticular plate. However, we did not observe continuous extensions of microtubules from the IHC periphery to the center. Rather, the isolated zones of microtubules after the onset of hearing suggest a *de novo* stabilization of microtubules at the center of the cuticular plate. Interestingly, a gain-of-function variant in *DIAPH1* gene, responsible for macrothrombocytopenia and hearing loss, increases actin polymerization and stabilizes microtubules (Ercan-Sencicek et al., 2015). It is therefore tempting to propose a similar scenario in this form of hearing impairment, i.e., an alteration of the apical pole of the hair cells. Consistently, transgenic mouse model of DFNA1 shows sparse and fused stereociliary bundle (Ueyama et al., 2016).

### Diap3 overexpression alters IHC mechanotransduction

Up to now, defective transmitter release is the most prevalent mechanism to cause human auditory neuropathies (Rance and Starr, 2015). However, synaptic recycling and the number of ribbon synapses were not affected in the diap3-overexpressing mutants, which excludes the loss of the ribbon synapses as a primary cause of AUNA1 deafness, as previously described (Schoen et al., 2013). In contrast, the major abnormality lies at the IHC apical side, with a distortion of the cuticular plate followed by the fusion of the stereociliary bundle. Although such abnormalities would most probably impair the activity of the mechanotransducer channel (Beurg et al., 2009), further experiments are needed to confirm whether the loss of the cuticular plate integrity affects the operating range of the mechanotransducer current (Fettiplace and Kim, 2014). However, the correlation between the degree of hearing loss to the anatomical features argues that the hearing impairment does not result from distortion of the cuticular plate but rather from the fusion of stereocilia. How can these two events be related to each other? Because the rootlets emanating from the stereocilia are embedded within the cuticular plate (DeRosier and Tilney, 1989; Furness et al., 2008; Vranceanu et al., 2012), loss of the anchoring structure might impede the stability of the stereocilia and lead to their collapse (Liberman, 1987; Kitajiri et al., 2010). Another cause of the severed stereocilia can be due to the dense microtubule matrix at the base of the stereocilia, which could interfere with key components of trafficking and delivery toward the stereocilia (Schneider et al., 2002; Zhang et al., 2012; Drummond et al., 2015). Finally, the invasion into the stereociliary bundle by microtubules at a later stage may disorganize their structure and lead to their fusion. Microtubules in the stereocilia might either come from the cuticular plate pool or be stabilized within the stereocilia. In the latter scenario, the activity rate of diap3 in the stereocilia should be slower than that of the cuticular plate, explaining the late onset of microtubule accumulation in the stereociliary bundle.

### IHC cuticular plate–specific alteration

Although diap3 is expressed in both OHC and IHC, only IHCs are affected. This can be explained by the lack of diap3 localization within the OHC cuticular plate, leading to spatial segregation of diap3 and the microtubule network in OHC. The discrepancy in diap3 distribution between hair cells might be due to a different rate of protein turnover. Indeed, the strong diap3 mRNA overexpression in the transgenic mice does not yield to an ubiquitous and massive distribution of diap3, suggesting a fine-tuning regulation at the protein level. The ubiquitination process may be more efficient in OHCs than in IHCs. In this hypothesis, OHCs would easily eliminate any excess of diap3 localized within the cuticular plate in contrast to IHCs (DeWard and Alberts, 2009). Alternatively, the specific IHC alteration might be due to the preferential expression in IHCs of a diap3 activator (a Rho-GTPase family member) or effector (a cytoskeleton binding protein; Evangelista et al., 2003; Wallar and Alberts, 2003; Goode and Eck, 2007; Bartolini and Gundersen, 2010). However, the known activators/effectors of diap3 are ubiquitously expressed. On the other hand, diap3 is highly regulated (Wallar and Alberts, 2003). Thus, a unique suppressor/inhibitor present in OHCs and not IHCs may leave OHCs unaffected. Therefore, we cannot definitively exclude the presence of unknown protein partners, exclusively expressed in one of the hair-cell populations, that modulate diap3 or undergo a diaphanous-dependent regulation. Finally, a difference in actin or microtubule metabolism in OHC versus IHC might also explain the difference of phenotype between the two hair cells.

### Diap3-overexpressing mice replicate human AUNA1 hearing loss

To decipher the mechanism of the auditory neuropathy AUNA1, caused by the overexpression of the formin protein diap3, we further investigated diap3-overexpressing Tg mice (Schoen et al., 2013). Our results demonstrate that these mutants replicate to some extent the AUNA1 features, i.e., a progressive hearing loss leaving OHCs largely unaffected. A more rapidly progressive threshold shift and a slight reduction of the DPOAE amplitude were observed in the 771 mouse line, which can be attributed to the morphological alteration and loss of a larger fraction of OHCs in this line. The different degree of OHCs vulnerability between the 924 and 771 lines might be due to the different number of transgene copies and thus in the diap3 expression between both genotypes. Thus, beyond a level of protein expression, the anatomical defects would not only be restricted to IHCs but would also occur in OHCs, leading ultimately to the degeneration of these cells. However, the mRNA expression level of diap3 is similar in both genotypes (Schoen et al., 2013), challenging this hypothesis. Alternatively, the transgene insertion site might differ between the two lines, and thus have a different impact on the survival of OHCs in the two diap3-overexpressing lines. Therefore, further analysis using a mouse harboring the point mutation found in humans with AUNA1 deafness would be required to resolve these different hypotheses.

We identify diap3 as a novel component of the stereociliary bundle of IHCs and OHCs, and of the IHC cuticular plate. Identifying a new molecular component increases our understanding of IHC dynamics by demonstrating a novel mechanism of deafness in the collection of disorders known as auditory neuropathy. Deciphering the mechanism of AUNA1 deafness will be the first step to developing a treatment for patients affected with this form of deafness.

## Synthesis

Reviewing Editor: Piali Sengupta, Brandeis University Decisions are customarily a result of the Reviewing Editor and the peer reviewers coming together and discussing their recommendations until a consensus is reached. When revisions are invited, a fact-based synthesis statement explaining their decision and outlining what is needed to prepare a revision will be listed below. Note: If this manuscript was transferred from JNeurosci and a decision was made to accept the manuscript without peer review, a brief statement to this effect will instead be what is listed below.

N/A
